# Clinical Spectrum of Arrhythmogenic Entities in Spanish Children Carrying Deleterious *SCN5A* Variants

**DOI:** 10.3390/ijms27020880

**Published:** 2026-01-15

**Authors:** Estefanía Martínez-Barrios, José Cruzalegui, Maria Hidalgo-Sanuy, Andrea Greco, Sergi Cesar, Fredy Chipa, Nuria Díez-Escuté, Patricia Cerralbo, Irene Zschaeck, Fernanda Merchán, Sol Balcells Mejia, Josep Brugada, Oscar Campuzano, Georgia Sarquella-Brugada

**Affiliations:** 1 Pediatric Arrhythmias, Genetic Cardiology and Sudden Death, Institut de Recerca Sant Joan de Déu (IRSJD), Santa Rosa 39-57, Esplugues de Llobregat, 08950 Barcelona, Spain; josecarlos.cruzalegui@sjd.es (J.C.); andrea.greco@sjd.es (A.G.); sergio.cesar@sjd.es (S.C.); fredy.chipa@sjd.es (F.C.); nuria.diez@sjd.es (N.D.-E.); patricia.cerralbo@sjd.es (P.C.); irene.zschaeck@sjd.es (I.Z.); fernanda.merchan@sjd.es (F.M.); 2 Arrhythmia, Inherited Cardiac Diseases and Sudden Death Unit, Department of Cardiology, Hospital Sant Joan de Déu Barcelona, Passeig Sant Joan de Déu 2, Esplugues de Llobregat, 08950 Barcelona, Spain; 3 European Reference Network for Rare, Low Prevalence and Complex Diseases of the Heart (ERN GUARD-Heart), 1105 AZ Amsterdam, The Netherlands; 4 Statistical Advising Service, Fundació de Recerca Sant Joan de Déu, Santa Rosa 39-57, 08950 Esplugues de Llobregat, Spain; sol.balcells@sjd.es; 5 Centro de Investigación Biomédica en Red, Enfermedades Cardiovasculares (CIBERCV), 28029 Madrid, Spain; josep@brugada.org (J.B.); oscar@brugada.org (O.C.); 6 Arrhythmias Unit, Hospital Clinic, University of Barcelona, 08036 Barcelona, Spain; 7 Medical Science Department, School of Medicine, Universitat de Girona, 17003 Girona, Spain; 8 Institut d’Investigació Biomèdica de Girona (IDIBGI-CERCA), Parc Hospitalari Martí i Julià, Edifici M2, 17190 Salt, Spain; 9 Departament de Pediatria, Facultat de Medicina i Ciències Mèdiques, Universitat de Barcelona, 08007 Barcelona, Spain

**Keywords:** *SCN5A*, rare variants, genetics, genotype–phenotype correlation, pediatrics

## Abstract

Deleterious variants in *SCN5A* lead to a wide clinical spectrum that includes pathologies characterized by life-threatening cardiac events (CEs). In the pediatric population, early identification, management, and risk stratification of these pathologies are the main current challenges. This study analyzed a Spanish pediatric cohort (≤18 years) carrying rare *SCN5A* variants to explore genotype–phenotype correlations. A retrospective descriptive cohort study, including clinical, demographic, and genetic data of probands and their relatives, was conducted. Out of 100 children studied, 69 had definitively deleterious *SCN5A* variants (26 females, 38%; median age: 3 years, IQR 1–12). The main diagnoses were isolated Brugada syndrome (BrS) (31; 45%); isolated long QT syndrome type 3 (LQT3) (5; 7%); isolated progressive cardiac conduction disease (PCCD) (1; 2%); isolated familial atrial fibrillation (1; 2%); overlapping phenotypes (7; 10%) including: BrS-PCCD (2; 2.8%); BrS-LQT3 (1; 1.4%); premature ventricular contraction-dilated cardiomyopathy (1; 1.4%); BrS-LQT3-PCCD (1; 1.4%); BrS-PCCD-sick sinus syndrome (SSS) (1; 1.4%) and BrS-PCCD-SSS-familial atrial fibrillation (1; 1.4%). Of them, 13 (19%) patients presented with CEs (cardiogenic syncope, ventricular tachycardia/fibrillation, sudden cardiac arrest/death, and appropriate implantable cardio defibrillator shock). These findings underscore the utility of genetic testing for early diagnosis, risk stratification, and personalized management, enhancing preventive strategies for CE prevention in pediatrics.

## 1. Introduction

The *SCN5A* gene (ENSG00000183873, OMIM^®^: 600163, UniProtKB: Q14524) encodes the pore-forming ion-conducting alpha subunit of the main cardiac voltage-gated sodium channel (Na_v_1.5). This channel induces the cardiac sodium current (INa), which is responsible for the rapid depolarization of cardiomyocytes, allowing for their contraction and propagation of the cardiac action potential (AP) throughout the myocardium [1]. Deleterious variants in *SCN5A* have been associated with several inherited arrhythmogenic syndromes (IAS) with a high risk of malignant arrhythmias and sudden cardiac death (SCD) [2]. Currently, the main associated syndromes with *SCN5A* are Brugada syndrome (BrS; OMIM#611777); long QT syndrome type 3 (LQT3; OMIM#603830); idiopathic ventricular fibrillation (IVF; OMIM#603829); familial atrial fibrillation (FAF; OMIM#614022); familial progressive cardiac conduction defect (PCCD; OMIM# 113900); familial sick sinus syndrome (SSS; OMIM# 608567); and dilated cardiomyopathy (DCM; OMIM#601154). To date, more than 500 rare variants in *SCN5A* have been classified as pathogenic (P) and likely pathogenic (LP), following the American College of Medical Genetics and Genomics and the Association for Molecular Pathology (ACMG/AMP) guidelines [3]. In general, loss-of-function (LoF) variants in *SCN5A* are associated with BrS, IVF, SSS, and PCCD [4]. In contrast, gain-of-function (GoF) variants are associated with LQT3 [5]. Other entities, such as FAF, have been associated with both GoF and LoF [6]. In addition, multiple arrhythmic phenotypes and overlapping syndromes have been frequently reported [7,8]. This heterogeneity, together with incomplete penetrance and variable expressivity (hallmarks of IAS), makes clinical translation still a major challenge for specialists, especially in the pediatric population. Due to the high variability of *SCN5A*-associated phenotypes, the trend is toward gene variant–phenotype correlation rather than gene–phenotype correlation.

Here, we present a Spanish cohort of children carrying *SCN5A* deleterious variants, providing new insights into genotype–phenotype correlations (at a variant level) in both pediatric patients and their relatives. Our findings contribute to enhancing clinical translation, improving diagnosis, risk stratification, and personalized management in this population.

## 2. Results

### 2.1. Genetic Results

**Figure 1:**
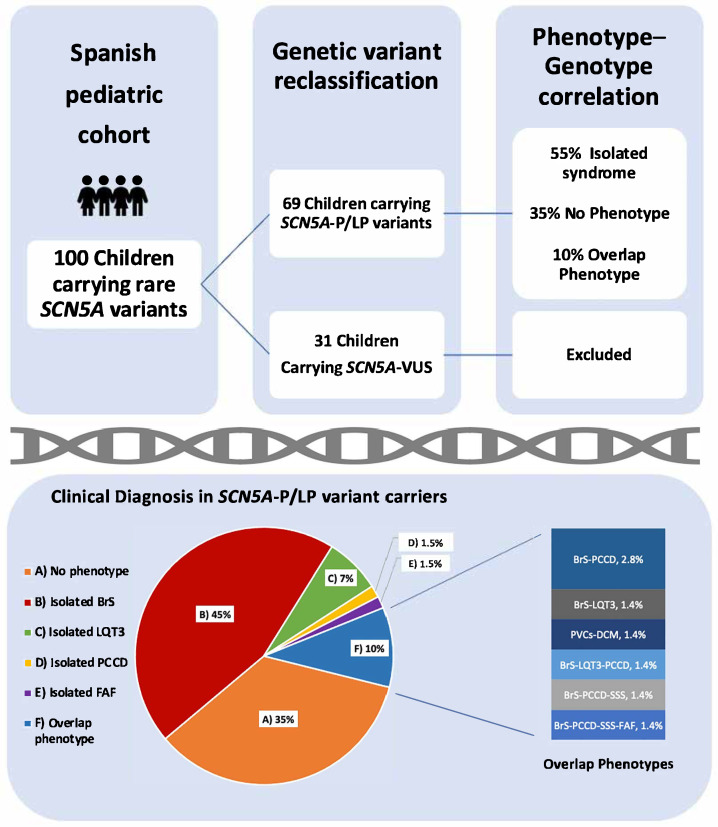
Inclusion criteria and phenotypes (clinical diagnosis) in children carrying *SCN5A*-P/LP variants. Abbreviations: P: pathogenic; LP: likely pathogenic; VUS: variant of uncertain significance; BrS: Brugada syndrome; LQT3: long QT syndrome type 3; PCCD: progressive cardiac conduction defect; FAF: familial atrial fibrillation; PVCs: premature ventricular contractions; DCM: dilated cardiomyopathy; SSS: sick sinus syndrome. Percentages in overlap phenotypes do not total 10% due to rounding.

**Figure 2:**
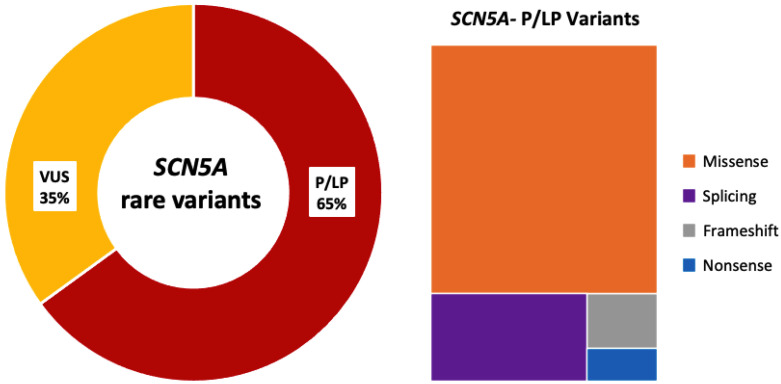
*SCN5A* rare genetic variants identified. Only definitive *SCN5A*-P/LP were included in this study. Abbreviations: VUS: variants of uncertain significance; P/LP: pathogenic/likely pathogenic.

**Figure 3:**
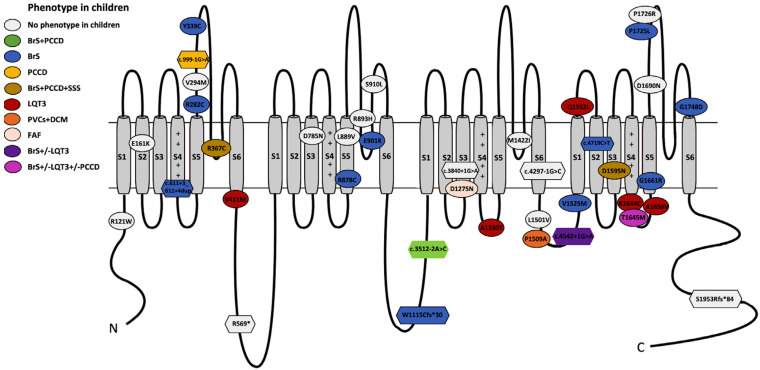
Genotype–phenotype correlation of *SCN5A* deleterious variants and localization along the Nav1.5 channel. Abbreviations: N: N-terminal; C: C-terminal; BrS: Brugada syndrome; LQT3: long QT syndrome; PCCD: progressive cardiac conduction disease; SSS: sick sinus syndrome; PVCs: premature ventricular contractions; DCM: dilated cardiomyopathy; FAF: familial atrial fibrillation. S1–S6 denote the six transmembrane segments of the channel protein. The positive charges on the S4 helices of each domain are indicated.

One hundred children from 79 families were analyzed. In total, 60 unique *SCN5A* variants were identified. All variants were re-assessed for the current study (February 2025; Supplementary Material, Table S1). In this reassessment, 13 of 34 variants initially classified as VUS were upgraded to P/LP (38%), and 21 remained as VUS (35%; 31 patients excluded from the genotype–phenotype correlation analysis) (Figure 1). In total, 39 unique variants were P/LP (65%), identified in 69 children, 12/39 pathogenic (P; 31%) and 27/39 likely pathogenic (LP; 69%); 29/39 were of the missense type (74%; in 42 children) and 10/39 truncating (26%; in 27 children, including splicing: 7/10; frameshift: 2/10; nonsense: 1/10) (Figure 2). Nine patients (9/69; 13%) carried two variants in *SCN5A*: 3/9 patients (33%) in the cis conformation and 6/9 patients (67%) in the trans conformation (compound heterozygous genotype). In 2/9 cases, the second variant was a P/LP variant, and in 7/9 it was a VUS. The distribution of the variants along the sodium channel is shown in Figure 3.

### 2.2. Descriptive Analysis of SCN5A-P/LP Carriers

A total of 69 pediatric patients below the age of 18 years and harboring a P/LP variant in *SCN5A* were eligible for the descriptive analysis (26 women, 38%; median age at diagnosis 3 years old—IQR 1–12). The median follow-up time was 5 years (IQR 2–9). Patients belonged to the Non-Finnish European (95%), Admixed American (3%), and African (2%) ethnic groups. The clinical characteristics of the cohort are listed in detail in Table 1. The description of the phenotypes associated with each of the *SCN5A*-deleterious variants, both in the children studied and in their relatives, is given in Table 2.

### 2.3. Family History

In total, 51/69 children had a family history of BrS (74%), 2/69 of LQT3 (3%), and 2 siblings had a family history of the mixed phenotype BrS+LQT3 (2/69; 3%). In total, 23/69 patients had a family history of SCD (33%). Only 8/69 children did not have any clinical family background (12%).

### 2.4. Clinical Diagnosis in Children Carrying SCN5A-P/LP Variants

In this cohort, 65% of the children (45/69) had a definitive clinical diagnosis. The majority showed an isolated syndrome (38/69; 55%). The most common isolated diagnosis was BrS, observed in 31/69 patients (45%), followed by LQT3 in 5/69 patients (7%), PCCD in 1/69 patient (2%), and FAF in 1/69 patient (2%). As shown in Figure 3, 7/69 pediatric patients (10%) exhibited an overlapping phenotype, including BrS-PCCD (2; 3%), SBr-PCCD-SSS-AF (1; 1.5%), BrS-PCCD-SSS (1; 1.5%), LQT3-BrS (1; 1.5%), BrS-LQT3-PCCD (1; 2%), and PVCs-DCM (1; 2%).

### 2.5. Clinical Findings in Patients with Isolated Syndromes

Among the 38 patients with a definitive isolated syndrome diagnosis, 14/38 (37%) were symptomatic. In the isolated BrS subgroup, 7/31 (23%) had symptoms, with 4/31 (13%) debuting during a febrile episode. Syncope was the most common symptom (6/31; 19%, including 3 febrile cases; 10%), followed by febrile seizures (FS; 3%) and epilepsy (3%). Febrile-induced arrhythmias (VF/VT) occurred in 2/31 (6%), while SCA (3%) and SCD before age 1 (3%) were also reported. Management included betablockers (6%), quinidine (3%), and implantation of an implantable cardioverter-defibrillator (ICD) in 6/31 (19%). In the isolated LQT3 subgroup, 2/5 (40%) were symptomatic, presenting with syncope (40%; 1 febrile), epilepsy (20%), and FS (20%). A total of 4/5 (80%) received betablockers, with quinidine added in 40%. One patient (20%) had severe LQT3, with prolonged QT, fetal bradycardia, VT/VF episodes, and torsade de pontes despite treatment (betablocker, quinidine, mexiletine, and left cardiac sympathetic denervation (LCSD)). This patient required ICD implantation before the age of 3 years and experienced multiple appropriate shocks during follow-up. The only patient diagnosed with isolated PCCD was symptomatic, had multiple syncope episodes, and required a PM implantation at 17 years of age. Finally, one patient with a long family history of FAF/SCD presented with asymptomatic isolated FAF/AF during follow-up and underwent effective ablation of the medial cavotricuspid isthmus.

### 2.6. Clinical Findings in Patients with Overlapping Phenotypes

Among the seven patients with overlapping phenotypes, five (71%) were symptomatic, presenting with severe phenotypes and CEs, including cardiogenic syncope (2/7; 29%), SCA (2/7; 29%), and SCD (1/7; 14%). The severe phenotypes of SCA were associated with BrS-PCCD and BrS-PCCD-LQT3, and one patient with PVCs-DCM experienced SCD at 18 years old. Management in this group included isolated antiarrhythmic therapy (57%), quinidine + beta-blocker combination (29%), ablation (40%), ICD implantation (40%), and PM programming (60%).

### 2.7. Genotype–Phenotype Correlation in Isolated Syndromes

Among the variants associated with isolated BrS in children, most were of the missense type (9/13; 69%, found in 13 BrS children), and 4/13 unique variants were truncating (31%, found in 19 patients, both splicing and frameshift). All variants associated with isolated LQT3 were of the missense type (4/4; 100%). Only one variant was found in a case of isolated PCCD and was of the splicing type. Finally, the missense variant p.D1275N was found in association with FAF in one patient.

### 2.8. Genotype–Phenotype Correlation in Overlapping Phenotypes

Seven children presented with overlapping phenotypes: 2/7 carrying the c.3512-2A>C showed BrS-PCCD; 1/7 presented with BrS-PCCD-SSS-AF carried the p.D1595N; 1/7 with BrS-PCCD-SSS carried the p.R367C and the VUS p.T1304M in trans; 1/7 showed the overlap phenotype BrS-PCCD-LQT3 and carried the variants p.T1645M and c.393-5C>A (VUS) in cis. The overlap phenotype BrS+LQT3 was observed in a patient carrying c.4542+1G>A. Finally, one patient carrying p.P1509A was found to show a particular phenotype with multiple PVCs, VF/AF, and SCD, with a post-mortem diagnosis of DCM.

## 3. Discussion

This study is one of the few to explore the broad spectrum of *SCN5A*-associated phenotypes in children carrying deleterious variants at the variant level, and the only one performed in the Spanish population. To ensure a precise genotype–phenotype correlation, we included only variants classified as P/LP. Cascade segregation analysis in adult relatives provided insights into phenotypic expression beyond pediatric age. Our cohort, with a very young mean age, had a high proportion of asymptomatic and undiagnosed patients, with only 65% of *SCN5A*-P/LP carriers exhibiting a definitive phenotype in childhood. However, at the family level, 100% of the identified variants correlated with a phenotype.

Clinical diagnosis in children carrying *SCN5A*-P/LP variants: In this cohort of children carrying *SCN5A*-P/LP variants, isolated BrS was the most common diagnosis (45%), aligning with the strong family history of BrS (74%) and SCD (33%). These findings are consistent with those of the multicenter study by Hermida, A. et al. in 2023 [9], which reported BrS in 54% of patients with *SCN5A*-P/LP variants, including both adults and children. However, they differ from the international registry presented by Baruteau et al., 2018 [10], which found only 2% of pediatric patients with BrS, likely due to the inclusion of VUS and variability in diagnostic protocols, particularly the limited use of sodium-blocking drug challenges in some centers. Other phenotypes in our cohort, though less frequent, included isolated LQT3 (7%), PCCD (2%), and FAF (2%). Complex phenotypes were also found in children carrying *SCN5A*-P/LP variants, consistent with previous studies [9,10], with 10% of patients exhibiting overlapping phenotypes, including BrS-PCCD, BrS-PCCD-SSS-AF, LQT3-BrS, BrS-LQT3-PCCD, and PVCs-DCM. In contrast to studies including adult populations, in which certain *SCN5A*-associated phenotypes are more prevalent in males than in females, this sex-based predominance was not observed in our pediatric cohort, in line with findings from other pediatric studies [10,11,12,13].

Clinical findings and genotype–phenotype correlation in children with isolated syndromes: Isolated BrS was the most prevalent phenotype. Among this subcohort, 69% of the identified variants were missense, all predicted to cause LoF, and were primarily located in the transmembrane extracellular portion of the channel (67%), with a significant clustering in domain IV (56%) and the voltage sensor region (67%). Previous studies indicated that variants in these regions frequently disrupt the voltage-sensing and gating mechanisms, leading to BrS phenotypes [14]. In contrast, 31% of variants in this subcohort were truncating (splicing or frameshift), responsible for 61% of pediatric BrS cases, reinforcing the higher penetrance of truncating *SCN5A* variants. Among children with isolated BrS, 23% exhibited symptoms, most commonly syncope (19%). Notably, 13% of BrS patients experienced their first symptoms during febrile episodes, supporting fever sensitivity in some *SCN5A*-BrS variants [15]. Specifically, in our cohort, two children (6%) presented with febrile-induced VF, and one suffered SCD during a febrile episode. This child harbored the splicing variant c.4719C>T in intron 27, predicted to cause mRNA degradation via nonsense-mediated decay (NMD). Functional studies confirmed that this variant results in the loss of the terminal 96 base pairs in exon 27, leading to complete INa abolition [16]. This was the most frequent variant in our cohort, present in 10 BrS-diagnosed children, predominantly fever-induced (4/10 showed type 1 BrS pattern during fever), and one exhibited an overlap phenotype (BrS-PCCD-SCA). Our findings suggest that fever can unmask or worsen BrS, increasing the risk of ventricular arrhythmias, particularly in cases involving FS. Therefore, careful fever management in children with BrS is crucial to mitigating these risks, especially in those with FS.

In the isolated LQT3 subgroup, all identified variants (100%) were missense and predicted to cause GoF, consistent with the pathogenic mechanism underlying LQT3 [17]. All were exclusively cytoplasmic, with no clear clustering at the protein level, suggesting diverse mechanisms of channel dysregulation. While hotspots associated with LQT3 have been proposed in the DIII-DIV interdomain (ID), pore, S4, and C-terminal region [18], no variants in these regions were detected in this cohort. The LQT3-associated variants identified in this pediatric cohort included the previously reported GoF, p.V411M, p.R1644C, and p.A1330T [19,20], and a novel de novo variant, p.A1656V. Within this subcohort, 40% of the patients presented with syncope, while 20% exhibited epilepsy and FS, highlighting the neurological overlap observed in some *SCN5A*-related LQT3 cases [21]. Notably, the patient carrying p.A1656V exhibited a severe neonatal phenotype, including 2:1 AVB (both prenatal and neonatal), extreme QT prolongation at birth (up to 840 ms), and life-threatening arrhythmias during febrile episodes. Management required a multimodal therapeutic approach, combining antiarrhythmic drugs, LCSD, and early ICD implantation with atrial pacing. At age 4, the patient developed epileptic seizures, likely associated with the neurological manifestations of the *SCN5A* variant, requiring dual antiepileptic therapy. This case further reinforces the importance of fever management in all *SCN5A*-P/LP carriers, including those diagnosed with LQT3, to prevent arrhythmic complications. Only one patient in our cohort presented with isolated PCCD; this patient harbored the splicing variant c.999-1G>A, reinforcing the previously reported link between truncating variants and PCCD [22]. This patient also carried the VUS *SCN5A*: p.T1304M in cis. To date, reported PCCD patients are mainly carriers of truncating variants or compound heterozygous/homozygous [23], although missense variants have also been reported. The missense p.D1275N variant was associated with isolated FAF in a pediatric patient. Located in the DII-S3 region of Nav1.5, it plays a role in the gating charge transfer center (GCTC) within the voltage-sensing domain (VSD). Though hypothesized to create a gating pore, its functional effects remain controversial [24]. Clinically associated with AF, PCCD, and DCM, the biophysical defects of this variant vary across experimental systems, showing reduced sodium current density, increased persistent sodium current, or altered voltage dependence. Further studies are needed to establish its definitive mechanism [25].

Overlap phenotypes in children: Four variants in our cohort were associated with overlapping phenotypes in children. In total, 75% were predicted to be LoF (50% missense and 25% splicing), and one missense was predicted to be GoF. The PCCD phenotype in our cohort was mainly observed as an overlapping phenotype, where most patients presenting with conduction disturbances also had other phenotypes associated with the *SCN5A* gene, such as BrS, LQT3, and SSS. Our findings indicate that the primary phenotypic overlap in pediatrics occurred between BrS-LQT3 and conduction diseases, aligning with previous studies across all ages, where conduction disturbances often represent the first manifestation of channelopathy [26]. In our cohort, we identified a girl with the BrS-PCCD-SSS-AF overlap phenotype, carrying the de novo *SCN5A* variant p.D1595N. This variant, previously reported in BAV, BRD, and AF, aligns with the observed phenotype. Functional studies have shown that p.D1595N enhances slower inactivation, reducing sodium channel availability and current density, contributing to conduction disease [27]. However, its specific role in the overlap phenotypes observed in the reported cases remains unknown. The splice LP variant c.3512-2A>C, located in intron 19 of *SCN5A*, was identified in two siblings with the BrS-PCCD overlap phenotype. Although previously unreported, it is predicted to cause LoF via NMD, aligning with the high prevalence of LoF variants in PCCD and BrS and the increasing recognition of their overlap [28]. Additionally, two siblings carrying the splicing variant c.4542+1G>A (intron 26) exhibited distinct phenotypes: one with BrS and the other with LQT3-BrS overlap. While this variant remains unreported and lacks functional studies, it is also predicted to lead to LoF through NMD. However, its mechanistic link to the BrS-LQT3 phenotype remains unclear. For other variants associated with BrS-LQT3, studies suggest a reduced peak current, a negative shift in steady-state INa channel inactivation, and an increased late INa current. This dual effect could explain QT prolongation via persistent inward INa current and BrS-like ST elevation via reduced peak INa current in epicardial cells. However, whether these specific mechanisms apply to this variant is still unknown [29].

Notably, in our cohort, p.P1509A was identified in a patient with multiple PVCs, AF/VF, and SCD at 18 years, which was associated with DCM in the postmortem analysis. This variant is reported for the first time in this study, and the pathophysiological mechanisms of the observed phenotypes remain to be determined. Although *SCN5A* is definitively associated with DCM, a deleterious *SCN5A* variant is found in only 1.7% of DCM families. Among them, the most common variant, p.R222Q, is well-characterized, exhibiting GoF effects on Na_v_1.5, increasing window current and premature action potentials in Purkinje cells, contributing to ventricular ectopy and progressive ventricular remodeling [30], a phenotype similar to that observed in our patient.

Overlap phenotypes in children with genetic heterogeneity: First, we highlight a case carrying two variants on different alleles of *SCN5A*, p.R367C (LoF-LP) and p.T1304M (VUS), who exhibited a BrS-PCCD-SSS phenotype. The p.R367C variant was established as the causative, having been previously reported in BrS, SCD, and conduction defects, as well as in both LQT3 and BrS, suggesting mixed-channel effects [31]. Located near the selectivity filter of the sodium channel, this variant may alter ion permeability and reduce INa [31]. p.T1304M, though associated with multiple cardiac phenotypes, has conflicting functional evidence [32,33]. While p.R367C is likely the primary driver of the phenotype, p.T1304M may act as a genetic modulator. Second, we identified two related patients carrying two variants with overlapping BrS-PCCD-LQT3 (1 patient) and isolated BrS (1 patient). Both children carried p.T1645M (LP-GoF), and c.393-5C>A was found on the same allele. p.T1645M has been reported in LQT3 cases [19] and is located in a mutation hotspot linked to severe cardiac phenotypes [34]. Interestingly, the adjacent p.R1644C has been associated with BrS-LQT3 overlap, suggesting that mutations in this region may contribute to dual phenotypes [33]. However, the c.393-5C>A variant classified as VUS, but was predicted to affect RNA splicing. A minigene RNA assay demonstrated exon 4 skipping, leading to a 30-amino acid loss in the DI transmembrane domain [35]. We hypothesize that the splicing variant could act as a genetic modulator in this family, explaining the variable phenotype observed in different members of this family. However, further studies are needed to establish a definitive mechanism.

Clinical and translational implications: Our findings emphasize the need for age-specific assessment in *SCN5A* variant carriers, particularly in early-onset cases and those triggered by febrile episodes, who can be at risk of CEs. Strict fever management and ECG during fever are recommended in all carriers of a P/LP variant, since fever, in addition to unmasking the phenotype, can act as a trigger for CEs. Several children required a sodium channel blocker challenge to reveal the diagnostic ECG, highlighting the utility of pharmacological provocation in diagnosing BrS in pediatric carriers of a deleterious variant in *SCN5A*. The heterogeneous presentation of *SCN5A*-related disorders, from silent carriers to severe arrhythmias, underscores the importance of comprehensive assessment and risk stratification in this population.

Limitations: Our study has limitations due to its observational and retrospective design, including information bias, as severe phenotypes were more thoroughly documented than asymptomatic *SCN5A* variant carriers. The limited sample size reflects the low global prevalence of these arrhythmogenic diseases, especially in pediatrics. In addition, functional data were not available for all identified variants, which limits the ability to assess their pathogenic mechanisms and biological impact.

## 4. Materials and Methods

### 4.1. Study Design

A retrospective cohort study was conducted with pediatric patients who were referred from 2015 to 2024 to the Arrhythmia, Inherited Cardiac Diseases and Sudden Death Unit, a reference center for pediatric arrhythmias in Spain. All patients aged below 18 years who were found to carry a rare *SCN5A* variant in the genetic testing were investigated, and those carrying a variant classified as P/LP following the ACMG/AMP criteria were included [3]. Patients carrying a P/LP in another gene, and those with incomplete data, were excluded.

### 4.2. Data and Variables

Demographic, clinical, and therapeutic variables were extracted from the electronic medical records. The clinical variables included the presence of symptoms (syncope, febrile seizures, epilepsy, etc.), basal ECGs, presence of arrhythmias, febrile onset, provocative pharmacological tests, ventricular arrhythmia inducibility in electrophysiology (EP) studies, final phenotype, and family history of IAS/SCD. All diagnoses were established following the 2022 ESC Guidelines [8]. The variables collected for the genetic analysis included the classification, type of variant, localization, and functional effect.

### 4.3. Genetic Analysis

Genomic DNA from the index cases was analyzed using next-generation sequencing (NGS), targeting 116 genes associated with IAS for comprehensive genetic evaluation (*ABCC9*, *ACTC1*, *ACTN2*, *AKAP9*, *ANK2*, *ANKRD1*, *BAG3*, *CACNA1C*, *CACNA2D1*, *CACNB2*, *CALM1*, *CALM2*, *CALM3*, *CALR3*, *CASQ2*, *CAV3*, *CRYAB*, *CSRP3*, *CTNNA3*, *GJA1*, *CTF1*, *DES*, *DMD*, *DMPK*, *DPP6*, *DSC2*, *DSG2*, *DSP*, *DTNA*, *ECE1*, *EMD*, *EN1*, *EYA4*, *FHL2*, *FKTN*, *FLNA*, *FLNC*, *GAA*, *GJA5*, *GLA*, *GPD1L*, *HCN2*, *HCN4*, *JPH2*, *JUP*, *KCNA5*, *KCND3*, *KCNE1*, *KCNE2*, *KCNE3*, *KCNE4*, *KCNE5*, *KCNH2*, *KCNJ2*, *KCNJ5*, *KCNJ8*, *KCNQ1*, *LAMA4*, *LAMP2*, *LDB3*, *LMNA*, *MYBPC3*, *MYH6*, *MYH7*, *MYL2*, *MYL3*, *MYLK2*, *MYOZ2*, *MYPN*, *NEBL*, *NEXN*, *NOS1AP*, *NOTCH1*, *NPPA*, *NUP155*, *PDLIM3*, *PHOX2A*, *PHOX2B*, *PITX2*, *PKP2*, *PLN*, *PRKAG2*, *RANGRF*, *RBM20*, *RYR2*, *SCN1B*, *SCN2B*, *SCN3B*, *SCN4B*, *SCN5A*, *SCN10A*, *SDHA*, *SGCD*, *SLC22A5*, *SLC6A4*, *SLC8A1*, *SLMAP*, *SLN*, *SNTA1*, *TAZ*, *TCAP*, *TGFB3*, *TLX3*, *TMEM43*, *TMPO*, *TNNC1*, *TNNI3*, *TNNT2*, *TP63*, *TPM1*, *TRDN*, *TRIM63*, *TRPM4*, *TTN*, *TTR,* and *VCL*). When the patients were not the index case, the specific variants were analyzed using the Sanger method. Analysis of copy number variations was performed using the Gendical Software version 2.1. Variants were reported according to the nomenclature recommended by the Human Genome Variation Society (HGVS). The prediction of the functionality was consulted using https://funnc.shinyapps.io/shinyappweb/ accessed on 5 March 2024. Variants were classified following the ACMG/AMP recommendations [3].

### 4.4. Statistical Analysis

A descriptive analysis was performed. Categorical variables were described using frequency tables, and numerical variables were described with mean (± standard deviation) or median [interquartile range (IQR)], depending on their distribution. Kolmogorov–Smirnov and Shapiro–Wilk tests were used to study the distribution of these numerical variables. Analyses were performed using IBM SPSS^®^ Statistics, version 30 (IBM Corp., Armonk, NY, USA).

## 5. Conclusions

Our study represents a large cohort of Spanish children carrying deleterious variants in *SCN5A* reported to date. This study provides relevant information on the genetic variants that may cause *SCN5A*-related phenotype/s in an age-evolving process, and cases of severe and/or overlapping phenotypes in pediatric ages. We emphasize the clinical utility of genetic testing in diagnostic strategies and the personalized management of the different phenotypes associated with *SCN5A* as a method that contributes not only to early detection and treatment but also to the adoption of preventive measures to reduce the risk of CEs.

## Figures and Tables

**Table 1:** Clinical and demographic data.

Children Carrying *SCN5A*-P/LP Variants	
	**n = 69 (%)**
**Demographic data**	
Sex. females	26 (37.7)
Age at debut or diagnosis (years)	3 (IQR: 1–12)
**Symptoms**	18 (26.1)
Syncope [Cardiogenic]	12 (17.4) [6 (8.7)]
SCD or SCA	5 (7.2)
Epilepsy	1 (1.4)
Cardiac event	13 (18.8)
**Response to fever**	
Debut with fever: symptoms or arrhythmias	8 (11.6)
Febrile syncope	3 (4.3)
Febrile seizures	5 (7.2)
Arrhythmias with fever	3 (4.3)
SCD/SCA associated with fever	3 (4.3)
**ECG Findings**	
Basal ECG abnormal	34 (49.3)
ECG abnormal during follow-up	47 (68.1)
QTc > 470 ms	7 (10.1)
Type 1 Brugada pattern [spontaneous/febrile]	36 (52.2) [9 (13)/10 (14.5)]
Type 2 Brugada pattern	4 (5.8)
Type 3 Brugada pattern	3 (4.3)
**Sodium channel blocker drug challenge**	35 (50.7)
Positive	27 (39.1)
**Arrhythmias**	
Bradycardia [sinus]	7 (10.1) [4 (5.8)]
AVB I-III	11 (15.9)
Atrial flutter/atrial fibrillation	5 (7.2)
Atrial Silence	1 (1.4)
VT/VF	9 (13.0)
**EPs for inducibility of ventricular arrhythmias**	13 (18.8)
Positive	1 (1.4)

Abbreviations: IQR: interquartile range; SCD: sudden cardiac death; SCA: sudden cardiac arrest; AVB: atrioventricular block; VT: ventricular tachycardia; VF: ventricular fibrillation; EP: electrophysiology study.

**Table 2:** Genetic variant–phenotype correlation in *SCN5A*-P/LP variant carriers.

*SCN5A* Nucleotide Variant (c.)	*SCN5A* Protein Variant (p.)	ACMG Classification	n (Pediatric Carriers)	Additional Variants	Phenotype in Pediatric Carriers	n (Adult Carriers)	Phenotype in Adult Carriers
c.361C>T	p.R121W	P	1	-	No	2	2 BrS
c.481G>A	p.E161K	P	1	-	No	3	2 BrS
c.611+3_611+4dup	p.?	P	6		4 BrS	7	6 BrS
c.844C>T	p.R282C	LP	2	-	1 BrS	3	1 BrS
c.880G>A	p.V294M	LP	2	-	No	1	BrS
c.999-1G>A	p.?	P	1	p.T1304M cis (VUS)	PCCD	2	1 SCD
c.1016A>G	p.Y339C	LP	1	-	BrS	1	BrS
c.1099C>T	p.R367C	P	2	1 p.T1304M trans (VUS)	1 BrS; 1 BrS-PCCD-SSS compound heterozygous p.T1304M (VUS)	1	BrS
c.1231G>A	p.V411M	P	1	-	LQT3	-	Unknown
c.1705_1706delCGinsTA	p.R569*	LP	2	-	No	1	BrS
c.2353G>A	p.D785N	LP	1	-	No	10	3 BrS
c.2632C>T	p.R878C	LP	2	-	2 BrS	3 BrS	1 BrS
c.2665C>G	p.L889V	LP	3	1 p.S524C trans (VUS)	1 BrS compound heterozygous with p.S524C (VUS)	2	No
c.2678G>A	p.R893H	LP	2	-	No	2	1 BrS
c.2701G>A	p.E901K	P	4	-	No	10	6 BrS
c.2729C>T	p.S910L	P	1	-	No	2	1 BrS
c.3345del	p.W1115Cfs*30	LP	1	-	BrS	1	BrS
c.3512-2A>C	p.?	LP	2	-	2 BrS-PCCD	4	4 BrS
c.3823G>A	p.D1275N	P	2	-	AF	2	1 BrS-FA
c.3840+1G>A	p.?	P	2	2 p.D1690N cis (P)	No	3	2 BrS
c.3988G>A	p.A1330T	LP	1	-	LQT3	1	No
c.4266G>A	p.M1422I	LP	1	-	No	4	4 BrS
c.4297-1G>C	p.?	LP	1		No	1	SCD
c.4501C>G	p.L1501V	LP	1	-	No	4	3 BrS
c.4525C>G	p.P1509A	LP	1	-	PVCs-DCM-SCD	2	1 BrS-SCD
c.4542+1G>A	p.?	LP	2	-	1 BrS-LQT3	5	1 Overlap BrS+LQT3, 1 isolated BrS, 1 isolated LQT3
c.4573G>A	p.V1525M	LP	1	-	BrS	3	3 BrS
c.4655A>T	p.Q1552L	LP	1	-	BrS	1	BrS
c.4719C>T	p.C1574_T1605del	P	10	1 p.D1819N cis (VUS)	9 BrS, 1 BrS-SCD	1	BrS
c.4783G>A	p.D1595N	LP	1	-	BrS-PCCD-SSS-AF (de novo)	-	NA
c.4930C>T	p.R1644C	LP	1	-	LQT3	1	LQT3
c.4934C>T	p.T1645M	LP	2	2 c.393-5C>A cis (VUS)	1 BrS-PCCD-LQT3	2	2 BrS-PCCD-LQTS in cis with c.393-5C>A (VUS)
c.4967C>T	p.A1656V	LP	1	-	LQT3 (de novo)	-	NA
c.4981G>A	p.G1661R	P	1	-	BrS	4	3 BrS
c.5174C>T	p.P1725L	LP	1	-	BrS	7	7 BrS
c.5177C>G	p.P1726R	LP	2	-	No	5	4 BrS, 1 SCD
c.5243G>A	p.G1748D	LP	1	-	BrS	1	BrS
c.5859_5862delTGAG	p.S1953Rfs*84	LP	1	1 p.A1870T trans (VUS)	No	1	BrS

The use of p.? is appropriate when the exact effect of the variant at the protein level cannot be precisely determined. Abbreviations: ACMG/AMP: American College of Medical Genetics and Genomics and the Association for Molecular Pathology; P: pathogenic; LP: likely pathogenic; BrS: Brugada syndrome; LQT3: long QT syndrome type 3; PCCD: progressive cardiac conduction disease; SSS: sick sinus syndrome; SCD: sudden cardiac death; SCA: sudden cardiac arrest; PVCs: premature ventricular contractions; DCM: dilated cardiomyopathy; AF: atrial fibrillation.

## Data Availability

All data generated or analyzed during this study are included in this published article.

## Supplementary Materials

The following supporting information can be downloaded at https://www.mdpi.com/article/10.3390/ijms27020880/s1.
